# Expression of bacteriocin divercin AS7 in *Escherichia coli* and its functional analysis

**DOI:** 10.1007/s13213-013-0759-x

**Published:** 2013-11-27

**Authors:** Agnieszka K. Olejnik-Schmidt, Marcin T. Schmidt, Anna Sip, Tomasz Szablewski, Włodzimierz Grajek

**Affiliations:** 1 Department of Biotechnology and Food Microbiology, Poznan University of Life Sciences, Wojska Polskiego Street No. 48, 60-627 Poznań, Poland; 2 Department of Food Quality Management, Poznan University of Life Sciences, Wojska Polskiego Street No. 31, 60-624 Poznań, Poland

**Keywords:** Bacteriocins, Divercin AS7, Human enterocytes, Biopreservatives

## Abstract

Bacteriocins are small peptides with antimicrobial activity, that are produced by bacteria. Four classes of bacteriocins produced by lactic acid bacteria have been defined. Class IIa bacteriocins are promising candidates for industrial applications due to their high biological activity and their physicochemical properties. Divercin AS7 is a class IIa bacteriocin produced by *Carnobacterium divergens* AS7. It shows antibacterial activity against pathogens and food spoilage flora including *Listeria* spp. Little is known about the impact of class IIa bacteriocins upon eukaryotic cells. The safe use of bacteriocins as food biopreservatives requires the absence of cytotoxicity to human cells. To analyze the impact of divercin AS7 on human enterocytes, we expressed the recombinant divercin AS7 in the *Escherichia coli* BL21DE3pLys strain and conducted in vitro studies to evaluate the safety of recombinant divercin AS7. No cytotoxic effect on differentiated monolayer Caco-2 cells and no apoptotic appearance were observed when recombinant divercin AS7 was used at a concentration of 2 μg ml^−1^. In our study, divercin AS7 also did not interfere with differentiated Caco-2 cells monolayer integrity. The obtained results suggest that divercin AS7 is a promising peptide for the food industry.

## Introduction

Bacteriocins are a heterogenous group of anti-bacterial ribosomally synthesized peptides. Lactic acid bacteria (LAB) produce bacteriocins that vary in their spectrum of activity and biochemical properties (Abee et al. 1995). Four classes of LAB bacteriocins have been defined on the basis of their structural characteristics. Class IIa bacteriocins are cationic heat-stable and membrane-active peptides containing disulfide bonds (Klaenhammer 1993; Nes et al. 1996). Bacteriocins could be useful as natural and non-toxic food preservatives. They can be produced in situ by bacteriocinogenic cultures or introduced to foods as additives (Abee et al. 1995). Moreover, some of them could be considered for other applications in human health (Todorov et al. 2010), and may also provide new approaches to combat antibiotic-resistant bacteria (Parisien et al. 2008). Divercin AS7 is a class IIa bacteriocin produced by *Carnobacterium divergens* AS7, a lactic acid bacterium isolated from fish. The amino acid sequence of divercin AS7 showed high homologies with divercin V41 and pediocin PA1. These bacteriocins contain two disulfide bonds responsible for their wider activity spectrum (Metivier et al. 1998). As its homologs, divercin AS7 shows antagonistic activity against closely related species, e.g., *Carnobacterium piscicola* (Sip et al. 1998) as well as food-borne pathogens, especially *Listeria* spp. (Lebois et al. 2004; Richard et al. 2003). Therefore, many studies are currently conducted to test the usefulness of divercin AS7 as a food or a feed additive (Jozefiak et al. 2011). Information is limited on the impact of class IIa bacteriocins upon eukaryotic cells, especially human enterocytes. To evaluate the safety of this kind of biopreservative, the cytotoxicity of purified bacteriocins should be determined. Studies on nisin used as food additive no. E234 (Directive 96/77/EC) shows contradictory data (Maher and McClean 2006; Murinda et al. 2003). It seems that the impact of nisin on humans cells is dependent on the type of cell and the amount of bacteriocin used in the test. Production of divercin AS7 by *Carnobacterium diverges* AS7 is very effective and optimized (Sip et al. 1998). The purification of this bacteriocin, as for other class IIa bacteriocins, is time-consuming and involves critical steps (Berjeaud and Cenatiempo 2004).

We used a heterologous expression system (*Escherichia coli*) to obtain bacteriocin divercin AS7 containing histidine-tag (His-Tag), purified recombinant protein and analyzed its impact on human intestinal cells Caco-2. This cell line is one of the in vitro systems most often used to mimic human intestine, and has the capacity to differentiate and express brush border enzymes (Di Cagno et al. 2010). These cells under culture conditions develop the morphological and functional characteristics of mature enterocytes. As far as we know, the presented work is the first to focus on the expression of a native gene encoding divercin AS7 to obtain a tagged protein in the *E. coli* heterologous system, and to test its functionality on human cells.

## Materials and methods

### Bacterial strains and media

*Escherichia coli* strain JM109 (Stratagene) was used for standard cloning procedures, and *E. coli* strain BL21(DE3)(pLysS) (Novagen) was used for gene expression experiments. *Escherichia coli* strains were grown aerobically in Luria-Bertani (LB) or SOC medium at 37 °C. Chemically competent cells and transformation of *E. coli* were prepared using standard procedures (Sambrook et al. 1989). The plasmid pET-28b+ (Novagen) was used for gene construction and expression. Transformants harboring pET28b+ were selected on LB agar medium containing 25 μg ml^−1^ kanamycin (for *E. coli* JM109) and LB agar medium containing 25 μg ml^−1^ kanamycin and 30 μg ml^−1^ chloramphenicol (for *E. coli* BL21DE3pLysS). *Carnobacterium divergens* AS7, a native divercin AS7 producer, was grown in MRS medium without Tween 80 at 30 °C and without shaking (De Man et al. 1960). *Listeria innocua* (ATCC33090), used as a divercin AS7-sensitive indicator microorganism, was grown in LZ medium at 37 °C without shaking.

### Recombinant plasmid construction

PCR primers used for this study were designed on the basis of DNA sequence for divercin V41 (AJ224003, GeneBank) and restriction enzymes sites for *Eco*RI and *Not*I were introduced into forward (5′-ATCAAGAATTCAACAAAATATTATGGGAAT-3′) and reverse (5′-ATTTTTGCGGCCGCTTAGCATTTACCTGGTA-3′) oligonucleotides, respectively. The oligonucleotides were purchased from the Institute of Biochemistry and Biophysics, Polish Academy of Sciences. The chromosomal DNA of *C. divergens* AS7 was used as a template in PCR amplification. The PCR reaction was conducted using 35 cycles of denaturing at 95 °C for 30 s, annealing at 54 °C for 30 s, and elongation at 72 °C for 1 min with BiometraT Gradient Thermal cycler. The amplicon was digested with *Eco*RI and *Not*I enzymes (Fermentas). The digested PCR fragment and linearyzed pET28b+ vector were purified from agarose gel using Gel-OUT Kit (A&A Biotechnology) according to the manufacturer’s recommendations. ORF AS7 was ligated into pET28b+ using T4 DNA ligase (Promega). Recombinant plasmid pET-AS7 was purified using Plasmid Mini Kit (A&A Biotechnology). The nucleotide sequence of the cloned DNA was analyzed using automated sequencing at the Institute of Biochemistry and Biophysics, Polish Academy of Sciences. The recombinant plasmid were verified by double digestion with *Eco*RI and *Not*I.

### Expression of divercin AS7 in *E. coli*

Overnight culture of *E. coli* strain BL21DE3pLysS harboring the recombinant plasmid pET-AS7 was diluted to 3 % (v/v) in SOC medium containing kanamycin (25 μg ml^−1^) and chloramphenicol (30 μg ml^−1^), and grown aerobically at 37 °C until the culture reached a density of 0.6 at 600 nm (measured in a spectrophotometer). Induction of gene expression was done by the addition of isopropyl-ß-D-thiogalactopyranoside (IPTG) to a final concentration of 0.1 mM. Expression was carried out for 24 h. At the specified time points (1, 2, 3, 4, and 24 h), 2 ml of culture was harvested by centrifugation (1,700*g*, 15 min, 4 °C). Bacterial biomass was stored at −80 °C.

### Protein expression analysis

Proteins were separated under denaturing and reducing conditions in 16.5 % Tricine-sodium dodecyl sulfate-polyacrylamide gel electrophoresis (Tricine-SDS-PAGE) (Schagger and von Jagow 1987), and transferred onto Immobilon-P membrane (0.2-μm pore size; Millipore) at 250 mA for 10 h in a buffer containing 25 mM Tris, 0.1 % (w/v) SDS, 192 mM glycine, and 20 % (v/v) methanol pH 8.3 using a Hoeffer apparatus (Amersham Pharmacia). After soaking in 100 % methanol for 30 s, the membrane was dried for 30 min and then saturated with 1 % (w/v) BSA in 1× TBST buffer (150 mM NaCl, 10 mM Tris–HCl pH 7.5, 0.1 % Tween-20). Then, it was incubated at room temperature for 30 min with an anti-HisTag mouse monoclonal antibody (Merck) diluted 1:1,000 (v/v) in 1× TBST buffer. After three washes with 1× TBST, the membrane was incubated at room temperature for 30 min with goat anti-mouse immunoglobulin G (biotinylated) diluted 1:5,000 (v/v) in 1× TBST. The membrane was washed three times with 1× TBST, then incubated at room temperature for 30 min with streptavidin peroxidase in TBST buffer. The substrate DAB was used to develop color reaction onto the membrane.

### Protein minipreps of recombinant divercin AS7 from *E. coli* under native conditions

Five milliliters of cell pellets resuspended (0.2 g ml^−1^) in buffer containing 50 mM NaH_2_PO_4_ 300 mM NaCl, and 10 mM imidazole, pH 8.0. The cells were disrupted by sonication in ice-cold water until the required visual viscosity was obtained. The separation of the cytoplasmic soluble fraction from cell debris was performed by centrifugation (14,000 *g*, 15 min, 4 °C). Recombinant divercin AS7 was purified using Ni-NTA agarose beads (Qiagen) according to the manufacturer’s recommendations.

### Bacteriocin activity assay

The bacteriocin activity was determined by the agar diffusion test (Richard et al. 2004). Antimicrobial activity of the recombinant divercin AS7 was investigated against *L. innocua* (ATCC 33090). An overnight culture of *L. innocua* in LZ broth (37 °C) was diluted in sterile LZ broth to 10^6^ CFU ml^−1^ before use. Drops (20 μl) of protein suspensions prepared under native conditions were placed on the top of the indicator strain-containing medium. The growth inhibition of indicator strain was determined after 24 h of incubation at 30 °C by observing the formation of inhibition zones.

### Cell culture

Human colon adenocarcinoma Caco-2 cells (ECACC, Cat. No. 86010202; Sigma-Aldrich) were grown in a controlled atmosphere of 5 % CO_2_ at 37 °C in Dulbecco’s modified essential medium (DMEM) containing 4.5 g l^−1^ glucose, 2 mM glutamine, 50 μg ml^−1^ gentamycin, 1 % non-essential amino acids, and 10 % heat-inactivated fetal calf serum. Experiments were performed using cells between passages number 49 and 52.

### Cytotoxicity assay

Caco-2 cells were seeded at a density of 10^5^ cells per well in 24-well culture plates (Greiner). Confluent cells were then exposed to divercin AS7 for 24 h. At the end of incubation, the effect of the bacteriocin on the plasma membrane integrity of Caco-2 cells was estimated by quantification of adenylate kinase release using the ToxiLight Non-destructive Cytotoxicity Bioassay Kit (Lonza). The percentage of cytotoxicity was calculated following the manufacturer’s instructions.

### Apoptosis assay

Caco-2 cells were cultivated in 24-well culture plates and incubated for 8 and 24 h with divercin AS7. The occurrence of apoptosis was evaluated by the method of DNA laddering (Barry et al. 1990). The supernatant and monolayers were collected and combined into a single sample. Caco-2 cells were washed with PBS and resuspended in lysis buffer. DNA was isolated using the Genomic Mini DNA purification kit (A&A Biotechnology) according to the manufacturer’s instructions. The DNA samples were separated by electrophoresis at 100 V for 45 min in 1 % (w/v) agarose containing ethidium bromide in 1× TBE buffer and visualized with UV light.

### Epithelial integrity

The effect of divercin AS7 on intestinal epithelial integrity was assessed by the measurement of transepithelial electrical resistance (TEER) (Klingberg et al. 2005). Caco-2 cells were seeded onto Millicell cell culture inserts (PTFE membrane, 0.4 μm pore size; Merck-Millipore, Poland) at 4 × 10^5^ cells cm^−2^ and cultured for 20 days to obtain a differentiated monolayer with media changes every 2 or 3 days. TEER was measured using a Millicell-ERS volt-ohm meter (Millipore), after 24 h of incubation of the Caco-2 cells with divercin AS7. The resistance of the monolayer was calculated by subtracting the intrinsic resistance of the system (filter insert alone) from the total resistance (monolayer plus filter insert). The data were displayed as percent TEER relative to the control at time zero.

## Results

### Production of recombinant divercin AS7 in *E. coli*

**Fig. 1:**
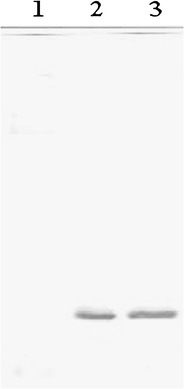
Western blot analysis for recombinant bacteriocin AS7 expressed in *E. coli*/pET-AS7 strain. The cells expressing bacteriocin AS7 were cultivated in SOC medium at 37 °C. Bacterial biomass before IPTG-induction (*1*) and after IPTG-induction 1 h (*2*) and 2 h (*3*) were subjected to western blot analysis. Bands (∼8 kDa) were detected using the anti-HisTag antibody

**Fig. 2:**
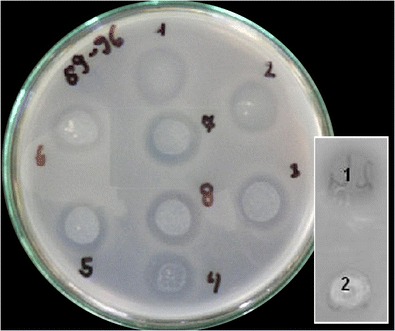
The agar diffusion test for recombinant bacteriocin AS7. *E. coli*/pET-AS7 lysates (20 μl) prepared from IPTG-induced culture after 2 h (1–8) were plated on LZ agar inoculated with *Listeria innocua* indicator strain and incubated overnight at 30 °C. Growth inhibition zones indicate action of recombinant bacteriocin AS7. The *inset* shows positive control (*1* diluted native bacteriocin AS7) and *E. coli*/pET-28b+ lysate (*2* negative control)

The expression of divercin fused to His-tag in *E. coli* BL21DE3pLysS/pET-AS7 was induced by the addition of IPTG. Tricine-SDS-PAGE and western blotting analysis confirmed the expression of fusion protein (Fig. 1). The molecular mass of recombinant divercin AS7 containing His-Tag is approximately 8.4 kDa (N terminal extension added due to construction requirements led to an enhancement of molecular mass). Maximum of expression occurs after 2 h post-induction. Analysis of the soluble and insoluble fractions, separated from induced *E. coli*/pET-AS7 cells, revealed that a fraction of the protein forms aggregates in the *E. coli* cell. Bacteriocin activity assay revealed that the soluble fraction of his-tagged divercin AS7 purified on Ni-NTA agarose beads retains its anti-*Listeria* activity (Fig. 2).

### Cytotoxicity assay

The cytotoxicity of divercin AS7 was analyzed on differentiated Caco-2 cells. For this purpose, the soluble fraction of divercin AS7 was tested at a concentration of 2 μg ml^−1^. The adenylate kinase released by the cells was quantified after 24 h of incubation. After 24 h of treatment of Caco-2 cells with divercin AS7, the percent of cytotoxicity remained at a background level. The observed adenylate kinase release was 2 % for 2 μg ml^−1^ of divercin AS7.

### Apoptosis assay

**Fig. 3:**
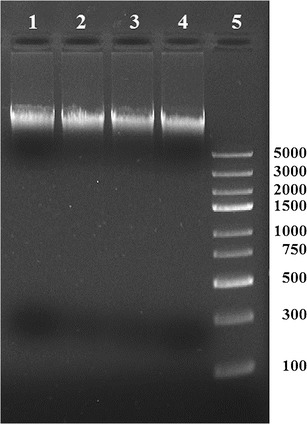
Agarose gel electrophoresis for DNA fragmentation assay (apoptotic ladder) showing Caco-2 genomic DNA isolated from untreated cells (*lane 1* negative control) and cells treated with bacteriocin AS7 for 1 h (*lane 2*), 4 h (*lane 3*) and 24 h (*lane 4*). *Lane 5* DNA ladder

DNA fragmentation is a common hallmark of apoptotic cells (Barry et al. 1990). DNA fragmentation assay was used to determine whether Caco-2 cells undergo apoptosis after incubation with divercin AS7. The quality of DNA isolated from Caco-2 cells after 24 h of treatment with 2 μg ml^−1^ of divercin AS7 was similar to DNA isolated from untreated cells (Fig. 3). No fragmentation (apoptotic ladder) was seen on the agarose gel, indicating that the divercin AS7 does not induce apoptosis of human intestinal enterocytes.

### Epithelial integrity

The effect of divercin AS7 on the epithelial monolayer integrity was studied on differentiated Caco-2 cells by measuring the transepithelial electrical resistance (TEER). After 24 h of treatment with divercin AS7 at 2 μg ml^−1^, no significant reduction in the TEER was observed. The average value of the TEER was 98.6 % of the initial level.

## Discussion

Bacteriocins have the potential for use as novel antibiotics and, in combination with traditional methods, as food preserving agents. To develop bacteriocins for these applications such as are needed for structural and functional studies, it is necessary to produce active highly purified peptide. Generally, class IIa bacteriocins have been obtained through the growth of the natural producer and peptide purification using cation exchange and reverse phase chromatography (Berjeaud and Cenatiempo 2004) or by phase partioning (Metivier et al. 2000). Heterologous expression of peptides is an alternative with many advantages, e.g., controlled expression, ease of purification, production of recombinant bacteriocin with a fusion tag, or expression of truncated forms for further analysis. Heterologous expression of class IIa bacteriocins in *E. coli* has shown that recombinant bacteriocins can be produced in an active form (Ingham et al. 2005; Richard et al. 2004; Yildirim et al. 2007). Although some problems have been reported with sub-cloned native genes of bacteriocins (Richard et al. 2004), we decided to express divercin AS7 from a native gene cloned in commercially available plasmid vector pET28b+. In our study, we obtained active recombinant divercin AS7 as a fusion protein with His-Tag. Recombinant divercin AS7 maintains its antibacterial activity as demonstrated by the agar diffusion test using *L. innocua* as the indicator strain. The activity of homologs of this bacteriocin have been linked to the C-terminus and its membrane-penetrating function, therefore the tag in our recombinant protein is fused to the N-terminus of peptide. Plasmid stability was monitored at every step of the experiment (data not shown), confirming that our recombinant construct is stable consisting of an insert. Due the small size of bacteriocins, they are not easily visualized on polyacrylamide gels, therefore we used Tricine gel electrophoresis and commercially available antibodies to the fusion partner (His-Tag) to identify production of the fusion bacteriocin. We observed formation of insoluble aggregates of divercin AS7 in the *E. coli* cell, but the use of a lower concentration of induction factor (0.1 mM IPTG) enabled us to obtain a soluble fraction of the correctly folded peptide. Further study will be required to determine the relationship between IPTG concentration and the efficiency of active protein synthesis. Production of divercin AS7 using a heterologous host could be limited by the higher cost compared to the natural way, but the protein obtained could be highly purified and used for in vitro studies on human cells. Our studies on the bacteriocin divercin AS7’s influence on intestinal epithelial integrity and cytotoxicity have shown that, at the concentration used (2 μg ml^−1^), the recombinant protein is not cytotoxic to undifferentiated Caco-2 cells. Moreover, we have shown on differentiated Caco-2 cells that divercin AS7 did not change the permeability of reconstructed epithelium. No variation in TERR was observed between the control value and the TERR of divercin AS7-treated Caco-2 cells. In studies on bacteriocin plantaricin A, it was shown that this has the potential to protect the function of the human intestinal barrier (TERR increase). Plantaricin A also enlarged the Caco-2 cell viability and contributed to the prevention of cell damage (Di Cagno et al. 2010). In our study, the absence of an apoptotic effect in the DNA fragmentation assay, along with lack of cytotoxicity and no damage to cell permeability, allow us to conclude that recombinant divercin AS7 at the concentration tested is not toxic to enterocytes and is a promising peptide for the food and pharmaceutical industry.

## References

[CR1] Abee T, Krockel L, Hill C (1995). Bacteriocins: modes of action and potentials in food preservation and control of food poisoning. Int J Food Microbiol.

[CR2] Barry MA, Behnke CA, Eastman A (1990). Activation of programmed cell death (apoptosis) by cisplatin, other anticancer drugs, toxins and hyperthermia. Biochem Pharmacol.

[CR3] Berjeaud JM, Cenatiempo Y (2004). Purification of antilisterial bacteriocins. Methods Mol Biol.

[CR4] De Man JC, Rogosa M, Sharpe E (1960). A medium for the cultivation of lactobacilli. J Appl Microbiol.

[CR5] Di Cagno R, De Angelis M, Calasso M, Vincentini O, Vernocchi P, Ndagijimana M, De Vincenzi M, Dessi MR, Guerzoni ME, Gobbetti M (2010). Quorum sensing in sourdough *Lactobacillus plantarum* DC400: induction of plantaricin A (PlnA) under co-cultivation with other lactic acid bacteria and effect of PlnA on bacterial and Caco-2 cells. Proteomics.

[CR6] Ingham AB, Sproat KW, Tizard ML, Moore RJ (2005). A versatile system for the expression of nonmodified bacteriocins in *Escherichia coli*. J Appl Microbiol.

[CR7] Jozefiak D, Sip A, Rawski M, Rutkowski A, Kaczmarek S, Hojberg O, Jensen BB, Engberg RM (2011). Dietary divercin modifies gastrointestinal microbiota and improves growth performance in broiler chickens. Br Poult Sci.

[CR8] Klaenhammer TR (1993). Genetics of bacteriocins produced by lactic acid bacteria. FEMS Microbiol Rev.

[CR9] Klingberg TD, Pedersen MH, Cencic A, Budde BB (2005). Application of measurements of transepithelial electrical resistance of intestinal epithelial cell monolayers to evaluate probiotic activity. Appl Environ Microbiol.

[CR10] Lebois M, Connil N, Onno B, Prevost H, Dousset X (2004). Effects of divercin V41 combined to NaCl content, phenol (liquid smoke) concentration and pH on *Listeria monocytogenes* Scott A growth in BHI broth by an experimental design approach. J Appl Microbiol.

[CR11] Maher S, McClean S (2006). Investigation of the cytotoxicity of eukaryotic and prokaryotic antimicrobial peptides in intestinal epithelial cells in vitro. Biochem Pharmacol.

[CR12] Metivier A, Pilet MF, Dousset X, Sorokine O, Anglade P, Zagorec M, Piard JC, Marion D, Cenatiempo Y, Fremaux C (1998). Divercin V41, a new bacteriocin with two disulphide bonds produced by *Carnobacterium divergens* V41: primary structure and genomic organization. Microbiology.

[CR13] Metivier A, Boyaval P, Duffes F, Dousset X, Compoint JP, Marion D (2000). Triton X-114 phase partitioning for the isolation of a pediocin-like bacteriocin from *Carnobacterium divergens*. Lett Appl Microbiol.

[CR14] Murinda SE, Rashid KA, Roberts RF (2003). In vitro assessment of the cytotoxicity of nisin, pediocin, and selected colicins on simian virus 40-transfected human colon and Vero monkey kidney cells with trypan blue staining viability assays. J Food Prot.

[CR15] Nes IF, Diep DB, Håvarstein LS, Brurberg MB, Eijsink V, Holo H (1996). Biosynthesis of bacteriocins in lactic acid bacteria. Antonie Van Leeuwenhoek.

[CR16] Parisien A, Allain B, Zhang J, Mandeville R, Lan CQ (2008). Novel alternatives to antibiotics: bacteriophages, bacterial cell wall hydrolases, and antimicrobial peptides. J Appl Microbiol.

[CR17] Richard C, Brillet A, Pilet MF, Prevost H, Drider D (2003). Evidence on inhibition of *Listeria monocytogenes* by divercin V41 action. Lett Appl Microbiol.

[CR18] Richard C, Drider D, Elmorjani K, Marion D, Prévost H (2004). Heterologous expression and purification of active divercin V41, a class IIa bacteriocin encoded by a synthetic gene in *Escherichia coli*. J Bacteriol.

[CR19] Sambrook J, Fritsch EF, Maniatis T (1989). Molecular cloning: a laboratory manual.

[CR20] Schagger H, von Jagow G (1987). Tricine-sodium dodecyl sulfate-polyacrylamide gel electrophoresis for the separation of proteins in the range from 1 to 100 kDa. Anal Biochem.

[CR21] Sip A, Grajek W, Boyaval P (1998). Enhancement of bacteriocin production by *Carnobacterium divergens* AS7 in the presence of a bacteriocin-sensitive strain *Carnobacterium piscicola*. Int J Food Microbiol.

[CR22] Todorov SD, Wachsman M, Tome E, Dousset X, Destro MT, Dicks LM, Franco BD, Vaz-Velho M, Drider D (2010). Characterization of an antiviral pediocin-like bacteriocin produced by *Enterococcus faecium*. Food Microbiol.

[CR23] Yildirim S, Konrad D, Calvez S, Drider D, Prévost H, Lacroix C (2007). Production of recombinant bacteriocin divercin V41 by high cell density *Escherichia coli* batch and fed-batch cultures. Appl Microbiol Biotechnol.

